# Intelligent Standalone Eye Blinking Monitoring System for Computer Users

**DOI:** 10.16910/jemr.17.5.1

**Published:** 2024-12-06

**Authors:** Ahmad A. Jiman, Amjad J. Abdullateef, Alaa M. Almelawi, Khan M. Yunus, Yasser M. Kadah, Ahmad F. Turki, Mohammed J. Abdulaal, Nebras M. Sobahi, Eyad T. Attar, Ahmad H. Milyani

**Affiliations:** Department of Electrical and Computer Engineering, King Abdulaziz University, Jeddah, Saudi Arabia; Center of Excellence in Intelligent Engineering Systems (CEIES), King Abdulaziz University, Jeddah, Saudi Arabia

**Keywords:** eye, blink, computer, monitor, screen, computer vision syndrome, gaze, attention, reading, new media

## Abstract

**Purpose**: Working on computers for long hours has become a regular task for millions of people around the world. This has led to the increase of eye and vision issues related to prolonged computer use, known as computer vision syndrome (CVS). A main contributor to CVS caused by dry eyes is the reduction of blinking rates. In this pilot study, an intelligent, standalone eye blinking monitoring system to promote healthier blinking behaviors for computer users was developed using components that are affordable and easily available in the market.

**Methods**: The developed eye blinking monitoring system used a camera to track blinking rates and operated audible, visual and tactile alarm modes to induce blinks. The hypothesis in this study is that the developed eye blinking monitoring system would increase eye blinks for a computer user. To test this hypothesis, the developed system was evaluated on 20 subjects.

**Results**: The eye blinking monitoring system detected blinks with high accuracy (95.9%). The observed spontaneous eye blinking rate was 43.1 ± 14.7 blinks/min (mean ± standard deviation). Eye blinking rates significantly decreased when the subjects were watching movie trailers (25.2 ± 11.9 blinks/min; Wilcoxon signed rank test; p<0.001) and reading articles (24.2 ± 12.1 blinks/min; p<0.001) on a computer. The blinking monitoring system with the alarm function turned on showed an increase in blinking rates (28.2 ± 12.1 blinks/min) compared to blinking rates without the alarm function (25.2 ± 11.9 blinks/min; p=0.09; Cohen’s effect size d=0.25) when the subjects were watching movie trailers.

**Conclusions**: The developed blinking monitoring system was able to detect blinking with high accuracy and induce blinking with a personalized alarm function. Further work is needed to refine the study design and evaluate the clinical impact of the system. This work is an advancement towards the development of a profound technological solution for preventing CVS.

## Introduction

Eye blinking is an essential physiological function for protecting the eyes and preserving ocular health. Eye blinks maintain a vital tear film on the ocular surface. In between blinks, the tear film becomes thinner and non-uniform across the corneal surface (41; 44). Long durations between blinks may lead to the disruption of the tear film and introduce deviations in the eye’s optical system (15). The tear film is immediately restored after a blink. Therefore, the ability to maintain a healthy tear film depends heavily on the eye blinking rate (44).

The eye blinking rate is influenced by many factors, such as ambient lighting, gaze direction, mental activity, attention, and task difficulty (3; 7; 14; 16; 17; 18; 34). Multiple studies have investigated eye blinking rates at different conditions. Eye blinking rates were reported higher when subjects were engaged in conversations, compared to blinking rates while performing other tasks, such as reading, watching or waiting (11). Conversational eye blinking rates were reported at 10.5 - 32.5 blinks/min, which is much higher than blinking rates when reading in a reading posture (1.4 - 14.4 blinks/min) or in a primary gaze position (8.0 - 21.0 blinks/min) (1; 7; 11; 26).

Working on computers for long hours has become a regular daily routine for millions of people around the world (29; 32). The drastically high number of computer users has led to the increase of health issues related to prolonged computer use, such as eye strain, eye fatigue, eye dryness, eye irritation, headaches and blurred vision (2; 4; 26; 32). The group of eye and vision complications associated with prolonged computer use is defined as computer vision syndrome (4; 26). The primary causes of computer vision syndrome are ocular motor and ocular surface factors (4; 28). Ocular motor responses to computer screens, such as accommodation and vergence, appear to be similar to viewing printed materials (28). However, multiple studies have reported that changes in ocular surface, specifically dry eyes, is strongly associated with prolonged computer use (42, 43). Furthermore, reading on computer screens produced the highest disturbance to the tear film of ocular surfaces compared to other reading devices and control (38).

A main contributor to computer vision syndrome caused by dry eyes is the reduction of eye blinking rates (4). Previous studies have shown that eye blinking rates while working on computers were reduced to 30 - 40% of the spontaneous eye blinking rate (13; 31; 40). Patients suffering from computer vision syndrome caused by dry eyes due to decreased eye blinking rates are typically prescribed with lubricating eye drops to alleviate the symptoms of this syndrome (4). However, these eye drops can cause a decrease in overall visual acuity and many users were dissatisfied with the therapeutic effects of eye drops (4; 33). Another common treatment solution to computer vision syndrome is taking frequent breaks from working on a computer (4). However, this solution may not be practical for many workers due to the demanding nature of their work, which requires them to continue working without interruption.

Recent technological advancements provide a valuable opportunity to explore impactful solutions to prevent computer vision syndrome. A research group developed a wearable device that uses infrared (IR) sensing to continuously track eye blinking rates and triggers blinks by light flashes, physical taps or air puffs near the eye (9). The study concluded that intense air puffs near the eye were the most effective in triggering blinks with low distraction ratings. However, the continuous emission of IR light directly to the eye and frequent intense air puffs near the eye raise safety concerns for the long-term use of this device (20). Another research group designed wearable blink-sensing glasses using a pressure sensor (6). The pressure sensor was developed by the research group using novel flexible iontronic sensing (FITS) technology that is not currently available in the market. Blinking monitors using a web camera have been developed with screen notifications to trigger blinks (8; 10; 21). These blinking monitors utilize the processing power of the computer being occupied by the user and screen notifications that may interfere with the user’s work on the screen. Therefore, there is a need for a blinking monitoring system composing of easily available components that is not obtrusive to the user and does not interfere with the processing unit or screen display of the user’s computer.

In this pilot study, a standalone eye blinking monitoring system using a camera and multiple independent alarm modes (audible, visual and tactile) was developed to induce eye blinks for computer users. The system does not visually interfere with the user’s work or impede the user’s computer performance. Furthermore, the components of the monitoring system are affordable and easily available in the market. The hypothesis in this study is that the developed eye blinking monitoring system would increase eye blinks for a computer user. To test this hypothesis, the eye blinking monitoring system was evaluated on a group of subjects to determine the impact of the blinking monitoring system on blinking rates while the subjects performed different tasks on a computer.

## Methods

### Materials

**Figure 1.**
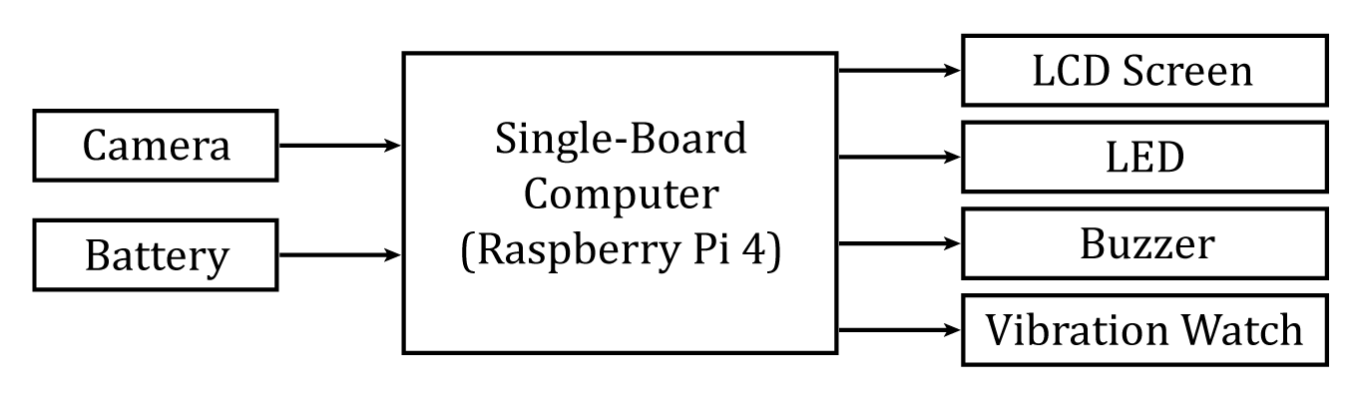
Block diagram of the eye blinking monitoring system.

**Figure 2.**
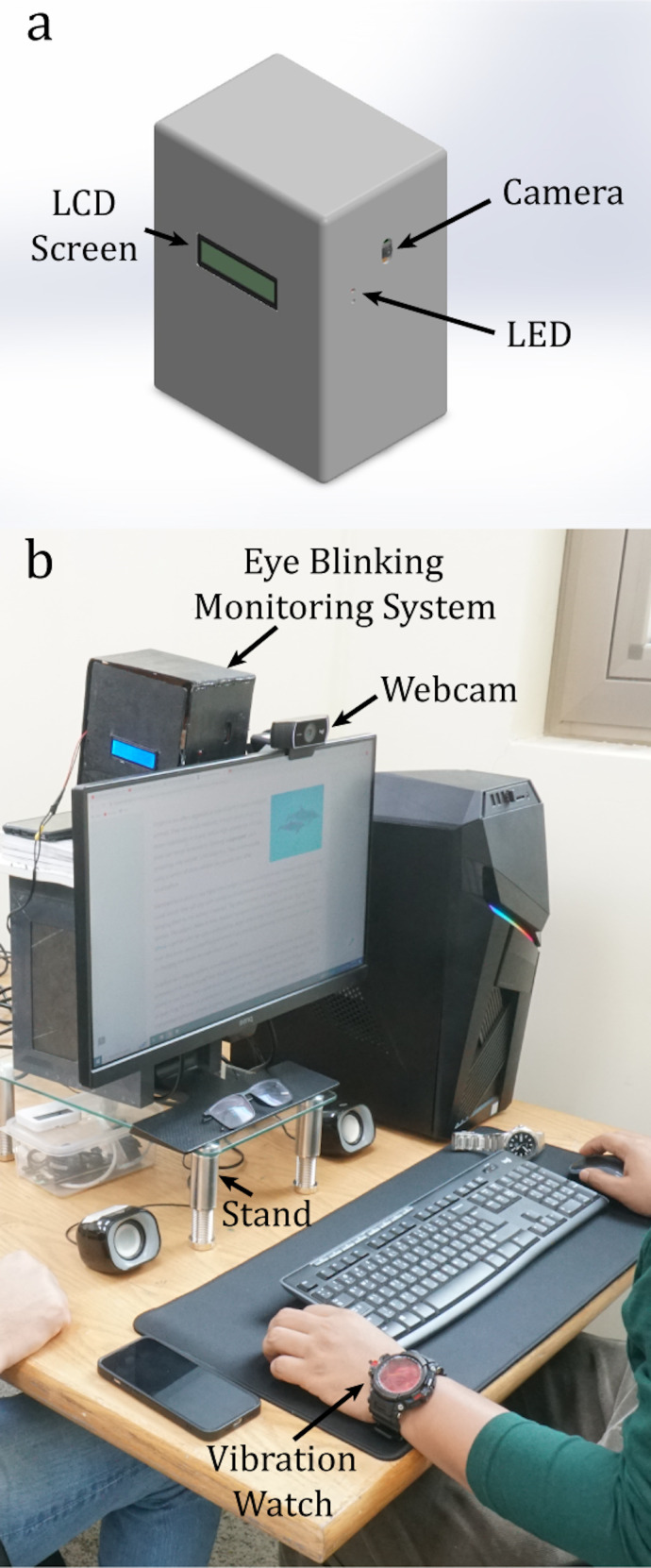
Eye blinking monitoring system and evaluation study setup. **a)** Design of the eye blinking monitoring system device. **b)** Experimental setup for the evaluation study.

The key components of the eye blinking monitoring system are a single-board computer (Raspberry Pi 4 Model B, Raspberry Pi, United Kingdom), camera (8 MP, Module 2, Raspberry Pi, United Kingdom), liquid crystal display (LCD) screen (I2C 1602, SunFounder, China) and battery power bank (10,000 mAh, Noon East, Saudi Arabia). The alarm modes for the eye blinking monitoring system are audible and visual notifications using a buzzer and light-emitting diode (LED), respectively. Another alarm mode is tactile vibration through a developed vibrating wristwatch that consists of a vibration motor and a small rechargeable battery. The wristwatch connects with the single-board computer using a radio frequency (RF) transmitter and receiver. The block diagram of the eye blinking monitoring system is shown in Fig. 1. The components of the developed system are easily available in the market with an affordable total cost of under $300. The components were assembled in a custom-built box with the dimensions of 14.5 x 10 x 19 cm (length x width x height). A small computer fan was added to the box to cool the electrical components. The design of the eye blinking monitoring system device is shown in Fig. 2a.

The main function of the eye blinking monitoring system is to continuously detect the user’s eyes and count the eye blinks. The developed programming code is a Python language script that uses OpenCV and Dlib libraries and Imutils package (27). The process of detecting and counting blinks is described in detail elsewhere (27; 35). Briefly, the camera captures video frames in real-time. These frames are processed and analyzed to identify facial features, particularly the eyes using the Haar cascade function for face detection and the Dlib shape predictor to identify faces within the video frames. These functions form boundary boxes around detected faces and extract facial landmarks. The facial landmarks serve as reference points for tracking eye movements. The distances between key points in the eye region are calculated, enabling the determination of blink status. The ratio of distances between vertical eye landmark points to the distances between horizontal eye landmark points are evaluated to assess whether a blink has occurred. If the ratio falls below a certain value, defined as Eye Ratio, a blink is registered (35). The Eye Ratio is determined by the user when the system is in Calibration Mode. In the event of a blink, the code enters a standby period of 0.5 sec before registering any other blinks to avoid false blink counts.

When the system is switched to Normal Mode, the code maintains a count of detected blinks during a specified time period determined by the user (set at 10 sec in this study). The blink count at the end of each time period is displayed on the LCD screen. If a face is not detected, the LCD screen will display the message “Face Not Found” and the time period for blink counts will not start until a face is detected. When the blink counts within the specified time period drops below a blinking threshold determined by the user, alarm notifications (buzzer sound, LED light and vibration of wristwatch) are triggered and a message “Please Blink More” is displayed on the LCD screen.

### Participants

The eye blinking monitoring system was evaluated on 20 subjects (13 men and 7 women) with a mean age of 26.7 years (age range: 18 - 60 years). The inclusion criteria were normal ocular health, age over 18 years, basic knowledge of computer use and the ability to read on a computer screen at a normal viewing distance without glasses. The number of participants in this pilot study was determined based on previous eye blinking monitoring studies for computer users (n=6 to n=24) (6; 8; 9; 21; 24; 26). The study protocol was approved by the Institutional Review Board at the Center of Excellence in Intelligent Engineering Systems at King Abdulaziz University. The study procedure and confidentiality measures were explained to the subjects before acquiring their written consent. A small gift (coffee coaster and chocolate) was given to each subject at the end of their participation in the study.

To assess the acceptance and usability of the developed eye blinking monitoring system, a modified online version of the System Usability Scale (SUS) questionnaire was sent to the subjects (5; 22). The SUS is a standardized questionnaire for the assessment of perceived user usability. The SUS consists of 10 items to be rated on a 5-point scale alternating between positive phrases (e.g. “I think that I would like to use the blinking monitor frequently.”) and negative phrases (e.g. “I thought the sound, light and vibration alerts of the blinking monitor were very disturbing.”). The final SUS score is computed based on the responses and the score ranges from 0 to 100 (22).

### Study Design

Each of the subjects in the study performed six tasks on a computer. The first task was designed to determine the optimal Eye Ratio for the subject. The aim of the second task was to determine the spontaneous eye blinking rate to set a personalized alarm threshold for each subject (50% of the spontaneous eye blinking rate). In the third and fourth tasks, the subjects watched movie trailers without and with the alarm of the eye blinking monitoring system, respectively. Similarly in the final two tasks, the subjects read articles on a computer without and with the alarm system.

The computer screen (22”, GW2280, BenQ, Taiwan) was placed on a stand to position the screen at a comfortable eye level for the subject at an approximate height of 55 cm. The viewing distance between the subject and the computer screen was around 60 cm. The blinking monitoring system was placed behind the computer screen with the camera slightly above the screen. A high-definition webcam (C922, Logitech, Switzerland) was placed on the computer screen to record the subjects during the study tasks and determine the actual blink counts. The webcam videos were recorded at a resolution of 720p and frame rate of 50 FPS. The study setup is shown in Fig. 2b.

### Procedure

Before the first task, subjects were instructed to blink at the sound of a metronome. This helped in anticipating the blinks to select the optimal Eye Ratio for each subject in Calibration Mode. Once the optimal Eye Ratio was determined, the subject performed the first task of blinking at the sound of a metronome at 36 BPM for 1 min, and at 42 BPM for another minute after a brief resting period. A timer with a beeping sound at the start and end of the 1-min period was used to synchronize between the blinking counts of the eye blinking monitoring system and the videos recorded by the webcam. For the remaining tasks, the system was switched to Normal Mode. In the second task, the subject answered to a set of verbal questions while gazing at the center of the computer screen for around 5 min to determine the spontaneous eye blinking rate. The natural variability in spontaneous eye blinking activity requires an observational period of 5 min or more to sufficiently determine the spontaneous eye blinking rate, which is a common guideline for studies that report spontaneous eye blinking rates (11). The eye blinking monitoring system alarm threshold was set at 50% of the spontaneous eye blinking rate. The subject was then requested to complete the third task of watching two movie trailers and answering a small set of questions after each movie trailer. The subject would then proceed to the fourth task that was similar to the previous task but with a different set of movie trailers and with the alarm of the eye blinking monitoring system turned on. After this, the subject was requested to take a break for at least 5 min away from the computer screen. When the subject was ready for the fifth task, the alarm system was turned off and the subject was instructed to read two articles with a few questions after each article. The subject would do the same in the final task but with a different set of reading articles and with the alarm system turned on. Each of the tasks with a set of two movie trailers or two reading articles were designed to be completed in approximately 5 min. The movie trailers selected for this study were for action movies to capture a subject’s attention without evoking emotional responses, such as laughter, which may cause facial expressions that impact the blink detection process (9). The selected reading articles in this study were scientific reading passages suitable for college students. The movie trailers and reading articles were randomized between the subjects to minimize the effect of confounding variables.

### Statistical Analysis

The blinking counts of the eye blinking monitoring system in the first task (blinking at the sound of a metronome) were compared to the true (actual) blinking events determined manually by watching the full length of the webcam video recordings played slowly at 0.5 speed. The error rate and accuracy were calculated using the following equations: Error Rate = | (Count – Actual) / Actual | x 100 and Accuracy = 100% – Error Rate, respectively. Furthermore, a Bland-Altman plot was constructed to display the relationship between the blink counts of the eye blinking monitoring system and the actual blink counts. The Bland-Altman plot shows the difference between the counted blinks by the eye blinking monitoring system and the actual blink counts over the average of both counts, with a bias line (mean difference) and 95% limits of agreement (mean ± 1.96 standard deviation). The number of alarm messages that occurred in the tasks with the alarm system of the eye blinking monitoring system turned on was determined by manually counting the alarm sounds that occurred in the recorded videos of these tasks or counting the registered blinking rates below the personalized threshold for a subject (videos of these tasks were not recorded for the first two subjects). The mean blinking rate was calculated for each subject and task. The differences between all the data sets that were used in statistical significance tests did not follow a normal distribution, which was confirmed by the one-sample Kolmogorov-Smirnov test. Therefore, a non-parametric Wilcoxon signed rank test was used to determine statistical significance, which was considered at *p* < 0.05. The statistical significance level was adjusted when performing statistical tests between multiple data sets using the Bonferroni correction method, where the significance level was divided by the number of tests. Cohen’s effect size (*d*) was calculated by dividing the difference between the two means over the pooled standard deviation of two data sets. The estimated median of the differences between two data sets was calculated with 95% confidence intervals (CI). All statistical analysis was performed using MATLAB software (R2023a, MathWorks, USA) and R statistical software (v4.4.1, R Core Team). When appropriate, values are presented as mean ± standard deviation.

## Results

The evaluation studies were performed on 20 subjects (13 male, 7 female) with a mean age of 26.7 years (age range: 18 – 60 years). None of the subjects had any known eye conditions during the study. Most of the subjects (12) did not normally were glasses or contact lenses, and 8 subjects normally wore glasses but they did not wear the glasses during the study tasks. The subjects were requested to remove their eye glasses for the consistency of the evaluation study. Removing eye glasses may have caused irritation for a few subjects but a subject remained in the same situation in all the tasks.

**Figure 3.**
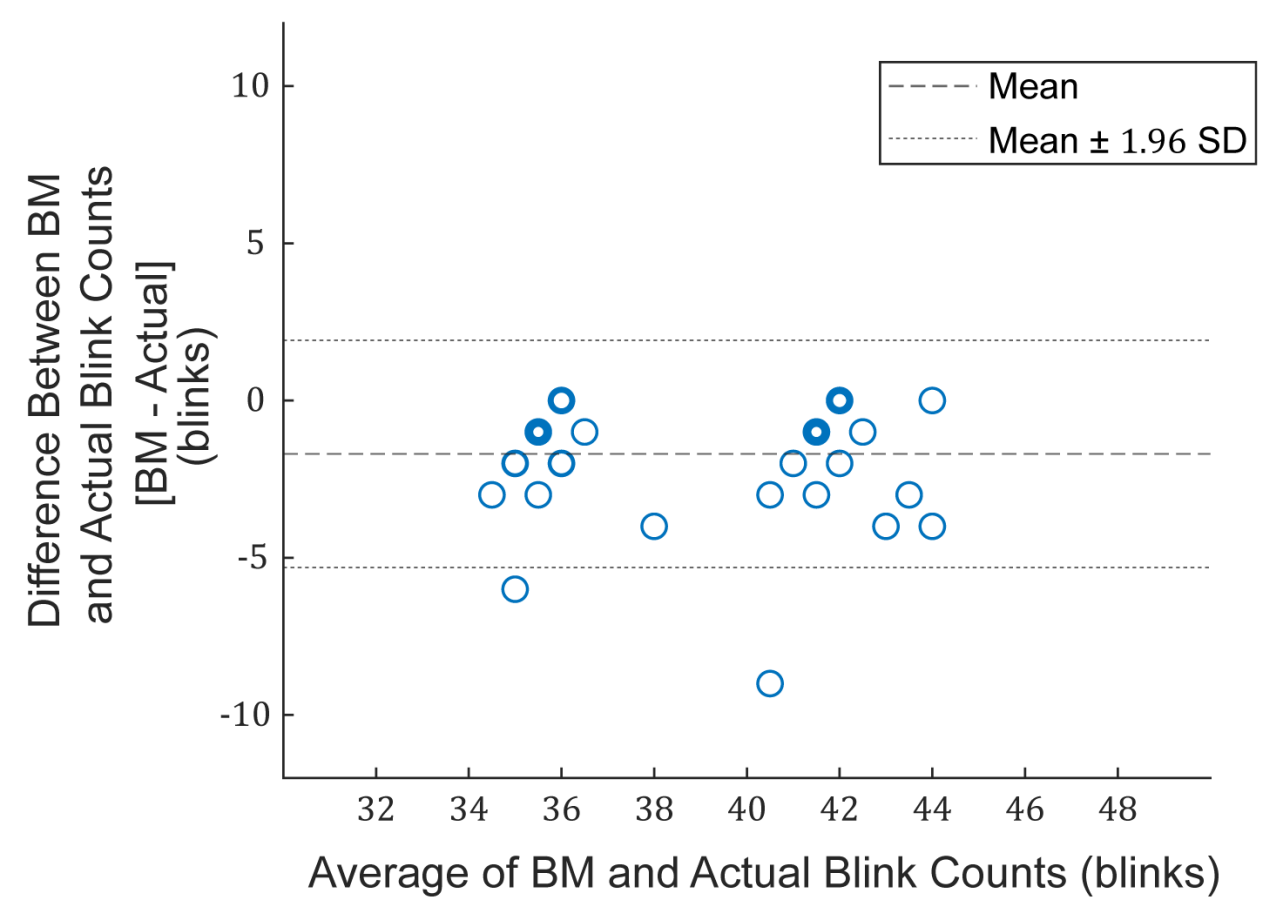
Bland-Altman plot for the blinking monitoring (BM) system and actual blink counts. The mean difference (bias) was −1.7 blinks. The negative sign indicates that on average, the blink counts of the blinking monitoring system was lower than the actual blink counts. The 95% limits of agreement (mean ± 1.96 standard deviation) were −5.3 and 1.9 blinks.

From the data obtained in the first task, the blinking counts of the eye blinking monitoring system were compared to the true blinking counts determined manually by watching the full-length of the recorded videos to calculate the error rate and accuracy. The overall mean error rate for the eye blinking monitoring system was 4.1 ± 4.1%. The accuracy of the eye blinking monitoring system was 95.9%. The mean difference between the blink counts of the blinking monitoring system and the actual blink counts (bias) was −1.7 blinks. The negative sign indicates that on average, the blink counts of the blinking monitoring system was lower than the actual blink counts. The 95% limits of agreement (mean ± 1.96 standard deviation) were −5.3 and 1.9 blinks. The Bland-Altman plot for the blinking monitoring system and actual blink counts is shown in Fig. 3. As expected, the data points in Fig. 3 are clustered around 36 and 42 blinks/min since the subjects were instructed to blink at the sound of a metronome at 36 and 42 BPM in the first task.

**Figure 4.**
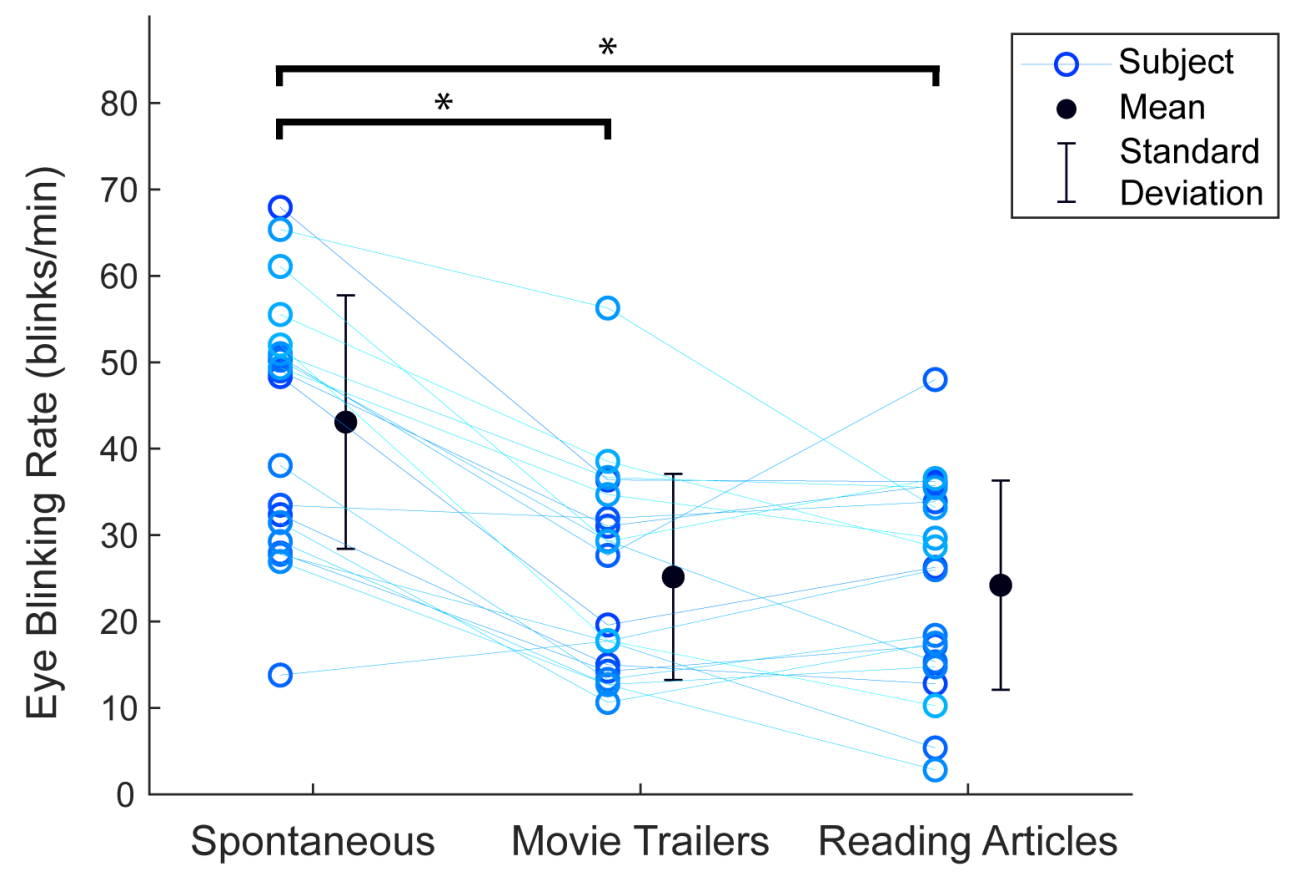
Eye blinking rates for all the subjects in a spontaneous (conversational) condition and tasks of watching movie trailers and reading articles without the alarm. Eye blinking rates for the subjects during the tasks of watching movie trailers and reading articles were significantly lower than spontaneous eye blinking rates (Wilcoxon signed rank test, α = 0.017 after Bonferroni correction, * = *p* < 0.001). The data points connected by a line are the blinking rates from a single subject.

The overall mean spontaneous (conversational) eye blinking rate for all the subjects was 43.1 ± 14.7 blinks/min. The eye blinking rate significantly decreased to 25.2 ± 11.9 blinks/min when the subjects were watching movie trailers (without the alarm) (*p* < 0.001, estimated median difference: −17.6 blinks/min, 95% CI: −22.9 to −14.0 blinks/min). A significant decrease to 24.2 ± 12.1 blinks/min was also observed when the subjects were reading articles (without the alarm), compared to the spontaneous eye blinking rate (*p* < 0.001, estimated median difference: −18.8 blinks/min, 95% CI: −24.3 to −13.2 blinks/min). There was no significant difference between the blinking rates of the subjects when watching movie trailers and reading articles without the alarm (*p* = 0.71, estimated median difference: −0.8 blinks/min, 95% CI: −5.2 to 4.0 blinks/min). The significance level was adjusted according to a Bonferroni correction in these tests (α = 0.05/3 = 0.017). The mean spontaneous eye blinking rate and blinking rates during movie trailers and reading articles for all the subjects are shown in Fig. 4.

The spontaneous eye blinking rate was calculated over a time period of 6.1 ± 1.4 min. Subjects completed the task of watching movie trailers in 4.4 ± 0.6 min without the alarm and 4.6 ± 1.9 min with the alarm (no significant difference, *p* = 0.49). The task of reading articles took 3.6 ± 1.8 min for the subjects to complete without the alarm and 2.9 ± 0.7 min with the alarm, with no significant difference (*p* = 0.33).

**Figure 5.**
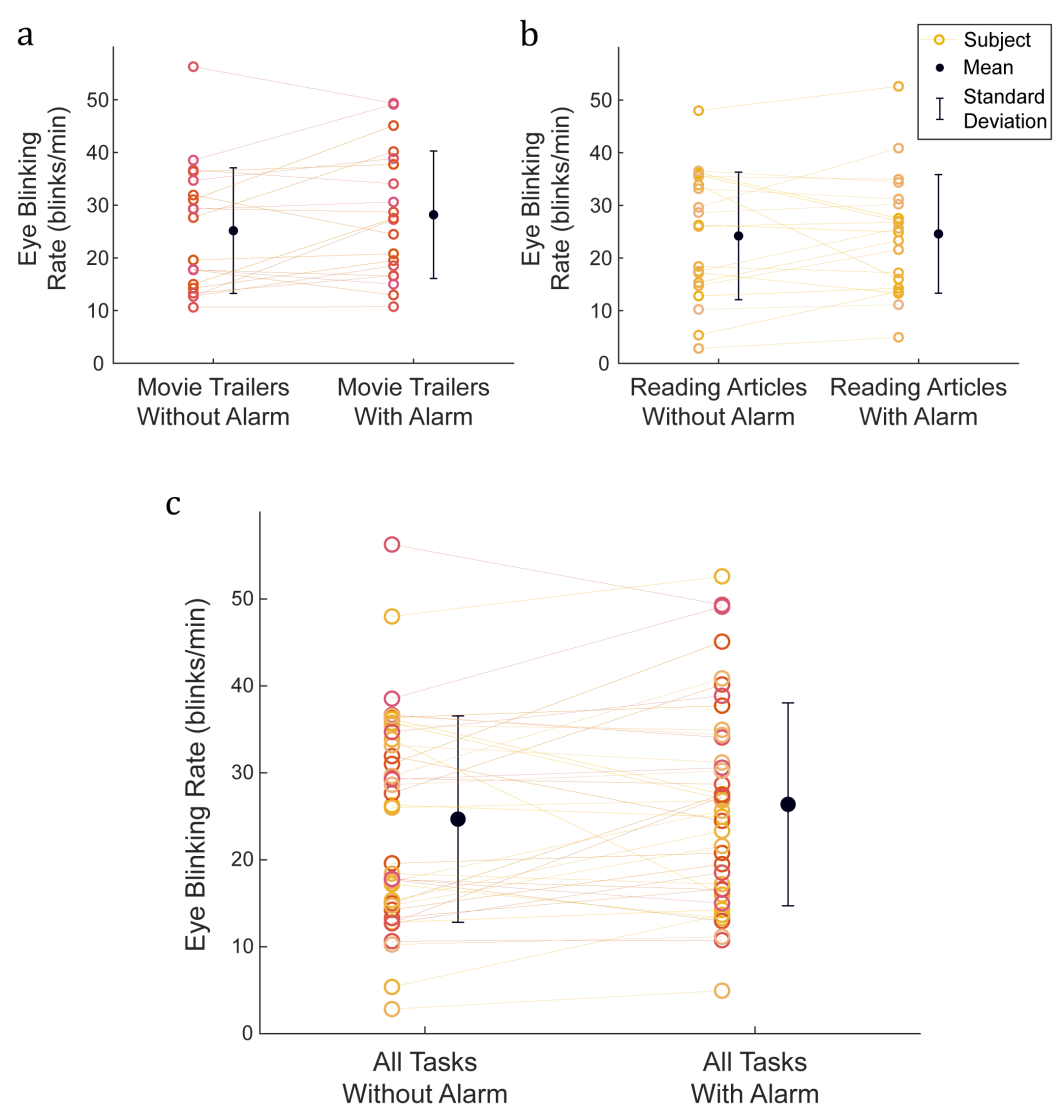
Effect of blinking monitoring system alarm on eye blinking rates. Eye blinking rates during the task of **a)** watching movie trailers, **b)** reading articles and **c)** both tasks without and with the alarm function. The data points connected by a line are the blinking rates from a single subject.

The threshold for the alarm of the eye blinking monitoring system was set between 1 and 6 blinks/10 sec (6 - 36 blinks/min), which was 50% of the spontaneous eye blinking rate for each subject. The number of alarm messages that occurred in the task of watching movie trailers with the alarm of the eye blinking monitor turned on was 9.0 ± 7.7 (range: 0 - 27) alarm messages, while 6.8 ± 5.7 (range: 0 – 20) alarm messages occurred in the task of reading articles. The overall mean blinking rate during the movie trailers without the alarm and with the alarm was 25.2 ± 11.9 blinks/min and 28.2 ± 12.1 blinks/min, respectively. The difference was not statistically significant (*p* = 0.09, estimated median difference: 2.8 blinks/min, 95% CI: −0.7 to 6.5 blinks/min) and the Cohen’s effect size (*d*) was 0.25. The mean blinking rate when the subjects were reading articles without the alarm was 24.2 ± 12.1 blinks/min, which was not statistically different than the mean blinking rate of 24.6 ± 11.3 blinks/min with the alarm (*p* = 0.68, estimated median difference: 0.5 blinks/min, 95% CI: −2.1 to 3.8 blinks/min, *d* = 0.03). The mean of all the blinking rates for all the subjects without the alarm during both tasks (watching movie trailer and reading articles) was 24.7 ± 11.9 blinks/min, while the mean of all the blinking rates with the alarm was 26.4 ± 11.7 blinks/min, with no statistically significant difference (*p* = 0.13, estimated median difference: 1.6 blinks/min, 95% CI: −0.4 to 4.0 blinks/min) and Cohen’s effect size of *d* = 0.14. The mean blinking rate for each subject while watching movie trailers and reading articles without and with the alarm is shown in Fig. 5. A summary for all the blinking rates observed in this study is shown in Table 1

An online System Usability Scale (SUS) questionnaire was sent out to the participating subjects and 17 subjects (85%) responded. The SUS score for the usability of the developed eye blinking monitoring system was 74.6 out of 100, which is a grade of B according to the Sauro-Lewis curved grading scale for general interpretation of SUS scores (22; 30).

**Table 1.** Summary of observed blinking rates in the evaluation study. Values are presented as mean ± standard deviation.

Condition	Blinking Rate Without Alarm (blinks/min)	*p*-value (vs Spontaneous)	Blinking Rate With Alarm (blinks/min)	*p*-value and Effect Size *d* (Without vs With Alarm)
Spontaneous (Conversational)	43.1 ± 14.7	-	-	-
Watching Movie Trailers	25.2 ± 11.9	*p* < 0.001	28.2 ± 12.1	*p* = 0.09, *d* = 0.25
Reading Articles	24.2 ± 12.1	*p* < 0.001	24.6 ± 11.3	*p* = 0.68, *d* = 0.03
All Tasks (Watching Movie Trailers and Reading Articles)	24.7 ± 11.9	-	26.4 ± 11.7	*p =* 0.13, *d* = 0.14

## Discussion

An intelligent, standalone eye blinking monitoring system for computer users was developed in this study. The developed system used a camera to track blinking rates and operated audible, visual and tactile alarm modes to induce eye blinks (Fig. 1 and 2). A personalized alarm threshold was set at 50% of the spontaneous (conversational) eye blinking rate for each user. The hypothesis in this study is that the developed eye blinking monitoring system would increase eye blinks for a computer user. The eye blinking monitoring system detected blinks with high accuracy (95.9%). A significant decrease was observed in blinking rates when subjects were watching movie trailers or reading articles on a computer, compared to spontaneous eye blinking rates (Fig. 4). The blinking monitoring system with the alarm function turned on showed an increase in blinking rates compared to blinking rates without the alarm function when watching movie trailers (Fig. 5), which supports the hypothesis. This work is an advancement towards the development of an effective technological solution for preventing computer vision syndrome to the highly prevalent and drastically increasing number of computer users around the world.

The eye blinking monitoring system successfully detected eye blinks with an accuracy of 95.9%. This accuracy level is similar to the accuracy levels of previous studies using infrared (IR) sensors (85.2%) (9), pressures sensors (96.3%) (6), cameras (85 - 97%) (8; 10; 12; 39) and microphones (95%) (23). The Bland-Altman plot in Fig. 3 showed that the eye blinking monitoring system on average underestimated the actual blink counts by 1.7 ± 1.8 blinks. The undetected blinks by the monitoring system were mostly involuntarily rapid consecutive blinks, which were the cause for the outlier data point observed in Fig. 3. The programming code of the monitoring system enters a standby period of 0.5 sec in the event of a blink before registering any other blinks. This standby period was introduced to avoid multiple counts for a single blink. However, this standby period needs to be investigated and adjusted in future studies to increase the detection accuracy of the eye blinking monitoring system. Another possible factor that may have caused the misdetection of blinks is the manual determination of the suitable Eye Ratio threshold for each subject. Eye Ratio is the ratio of distances between eyelid landmarks to assess whether a blink has occurred. The Eye Ratio was selected manually in this study but the development of an automated calibration process, including the selection of a suitable Eye Ratio for each user, is expected to considerably reduce the blink misdetections, and in return, increase the accuracy of the system. Furthermore, calculating the spontaneous eye blinking rate to determine the personalized alarm threshold for each individual could be integrated in the calibration process in future eye blinking monitoring systems to facilitate the setup process for a user.

The eye blinking rates in this study were noticeably higher than reported values in previous studies. The observed eye blinking rates of this study in a spontaneous (conversational) setting was 43.1 ± 14.7 blinks/min, which decreased to 25.2 ± 11.9 blink/min while watching movie trailers and 24.2 ± 12.1 blinks/min when reading articles at the primary gaze level. These values are marginally higher than the reported eye blinking rates in previous studies, which were 10.5 – 32.5 blinks/min during conversations, 12.5 – 20.0 blinks/min while watching movie trailers and 8.0 – 21.0 blinks/min in primary gaze positions (7; 9; 11). There are multiple possible reasons for the increased level of blinking rates in this study. It was noticed that the blinking monitor would count blinks continuously when the subject’s gaze position is much below the primary gaze level. Thus, a stand for the computer screen was added to the setup (Fig. 2b) that allowed the subjects to comfortably view the screen at their primary gaze level. It was also noticed in some occasions that the blinking monitor would count false blinks when the subject is laughing especially during the conversation task of the study. Differentiation between actual blinks and false blink counts as a result of facial expressions needs to be considered when developing an accurate blinking monitoring system. Another possible reason is the nervousness of the subjects during the study experiments. Studies have shown that nervous tension may lead to increased rates of blinking (25). Inserting an adaptation period in future blinking monitor evaluation studies may help in capturing blinking rates within the expected ranges reported in previous studies (7; 11; 13).

The study participants that completed the System Usability Scale (SUS) questionnaire graded the usability of the developed blinking monitor system at a score of 74.6 out of 100, which is considered generally good (B grade) according to the Sauro-Lewis curved grading scale for general interpretation of SUS scores (22; 30). The SUS score is more meaningful when compared to other eye blinking monitors but none have been reported so far. A major aspect of the usability and acceptance of a blinking monitor is the alarm system. The developed eye blinking monitoring system in this study used three different alarm modes to induce blinks: audible sound, visual light and tactile vibration. Most of the subjects found the system alerts not very disturbing. The item “I thought the sound, light and vibration alerts of the blinking monitor were very disturbing” was rated 1.7 out of 5 in the SUS questionnaire. Although all of these alarm modes were used simultaneously in this study to increase the impact an alert, the overall increase in blinking rate with the alarm system compared to without the alarm was only 6.9% with no statistical significance and an effect size (*d*) of 0.14, which is considered a small effect size (36). A more tailored approach based on the user’s preference is likely to be more effective and sustainable for inducing blinks. In previous studies, physical tapping near the eye was found to be an effective tactile alarm that resulted in a 35.8% increase in the participants’ mean blinking rate (9). Double blink animations on computer screens increased blinking rates by 91.8% (24). Slowly blurring a screen until the user blinked increased blinking rates by over 100% (8). The low increase in blinking rate observed in this study is likely due to the misdetection of blinks. In addition to the misdetection of rapid consecutive blinks due to the 0.5 sec standby period after each blink, it was noticed in later stages of this study that blinks were not being detected during the momentary alarm trigger period (~1-2 sec). In previous studies, participants blinked in response to 56.2% of the physical taps within the first 2 sec (9) and the average blinking response to a blurring screen was 1.7 sec (8). Therefore, this issue in the programming code has likely severely diminished the effect of the alarms in this study and must be addressed with high priority in future studies. Furthermore, no instructions were provided to the participants in this study on the appropriate response to an alarm. Instructing users to blink twice upon receiving an alarm may be an effective method to increase blinking rates with a lower number of alarm messages (24) and hence, increase the acceptance of a blinking monitor designed for daily use. More studies are needed to investigate tailored alarms and instructions to determine the most effective method for inducing blinks with minimal distraction.

There are some limitations to this study. The misdetection of rapid consecutive blinks and blinks during the alarm trigger period that were explained earlier are considerable limitations to this study and must be addressed. Although the subjects were requested to remove their eye glasses for the consistency of the evaluation study, the eye blinking monitoring system can detect eye blinks for subjects wearing eye glasses with reasonable accuracy (77.8%, n=1). More trials and modifications on the programming code are required to improve the accuracy of blink detection using cameras for users with eye glasses. Along with decreased eye blinking rates, higher percentages of incomplete eye blinks during computer use is considered another major possible contributor to the prevalence of computer vision syndrome (15; 26; 37). An exploration study showed that symptoms of computer vision syndrome for computer users was directly associated with blinking rate (21). The results of this study reported that for each added blink to the average blinking rate, the computer vision syndrome score decreased by 1.3. This suggests that even a small increase in blinking rate can have a positive impact on alleviating symptoms of computer vision syndrome. However, another study showed that the score of computer vision syndrome symptoms was associated with incomplete blinks, while an increase in blinking rate did not produce a significant change in the symptoms score (26). This conflict of results requests the detection of blinking rate and incomplete blinks in blinking monitor studies. Unfortunately, incomplete blinks were not assessed in this study due to limitations in developing the programming code but should be considered in future blinking monitor studies. Furthermore, obtaining clinical measurements, such as tear film parameters (e.g. tear meniscus height, tear break-up time, tear osmolarity and tear volume), pupil size to quantify visual fatigue and questionnaires to evaluate dry eye symptoms and computer vision syndrome symptoms are necessary to clinically evaluate blinking monitors and must be considered in future studies (19; 38). In the design of this study, training and fatigue are factors that may have had an effect on blinking rates. However, the training and calibration process (tasks 1 and 2) were reduced to the minimum possible time of around 10 min to mitigate potential fatigue at the end of the experiment. Also, after completing the tasks of watching movie trailers (tasks 3 and 4), the subjects were requested to take a break for at least 5 min away from the computer to reenergize themselves before proceeding to the tasks of reading articles (tasks 5 and 6) and completing the experiment. Although the movie trailers and reading articles selected in this study were randomized between the subjects to minimize the effect of confounding variables, the fixed order of the tasks (watching movie trailers and reading articles with and without the alarm of the eye blinking monitoring system) is a possible confounding variable. The order of these tasks should be randomized to reduce the effects of any potential confounding variables and must be considered in future blinking monitor studies. Also, some users may find difficulties with the current setup process for the developed eye blinking monitoring system, which was performed by the experimenters in this study. The setup process includes appropriate positioning of the blinking monitoring system, specifying the optimal Eye Ratio for each user, and setting the personalized alarm threshold for each individual based on their spontaneous eye blinking rate. More investigations are needed to determine the optimal setup process that can be performed by a user to operate an eye blinking monitoring system on a daily basis.

Overall, an intelligent, standalone eye blinking monitoring system for computer users was developed in this study. The experimental procedures showed that the developed blinking monitoring system was able to detect blinking with high accuracy and induce blinking with a personalized alarm function. The developed system is completely non-obtrusive for the user and independent of the user’s computer. The components of the developed system are affordable and easily available in the market. Further work is needed to avoid blink misdetections in specific conditions, assess incomplete blinks, refine the study design, evaluate clinical parameters and investigate effective alarm triggers for inducing blinks. This work provides a technological advancement for preventing eye and vision complications to the highly prevalent and drastically increasing number of computer users around the world.

### Ethics and Conflict of Interest

The authors declare that the contents of the article are in agreement with the ethics described in http://biblio.unibe.ch/portale/elibrary/BOP/jemr/ethics.html and that there is no conflict of interest regarding the publication of this paper.

### Acknowledgements

This project was funded by the Deanship of Scientific Research (DSR) at King Abdulaziz University, Jeddah, Saudi Arabia, under grant no. J: 11-135-1441. The authors gratefully acknowledge DSR for their technical and financial support. The authors thank the reviewers for their insightful comments and impactful recommendations.
